# Microbiota in Health and Disease

**DOI:** 10.3390/life15091374

**Published:** 2025-08-29

**Authors:** Amélia Sarmento, Catarina D. Simões

**Affiliations:** 1 RISE-Health, Faculty of Health Sciences, Fernando Pessoa University, Fernando Pessoa Teaching and Culture Foundation, Rua Carlos da Maia 296, 4200-150 Porto, Portugal; catarinasimoes@ufp.edu.pt; 2 Instituto de Investigação e Inovação em Saúde, Universidade do Porto, Rua Alfredo Allen 208, 4200-135 Porto, Portugal

## 1. Introduction

In recent years microbiota has been shown to strongly influence host metabolism and immunity, resulting in a huge impact in human physiology [1,2,3]. Understanding the role that different microbial groups and their metabolites play in modulating those processes is pivotal both for the opening of new perspectives towards disease prevention and for developing new and personalized therapies. In line with this, the main aim of the current Special Issue was to join different contributions addressing the state-of-the art research on human microbiota and its impact on human well-being or disease.

Fourteen contributions were included in this Special Issue. Contribution 1 evaluated various techniques for extracting DNA from fecal samples and breast tissue, as well as different sequencing approaches, with the aim of enhancing result accuracy by reducing the influence of human DNA contamination. Other contributions focused on the resilience and dynamics of gut microbiota (Contribution 2) and the interconnected axes with other organ systems, such as the skin (Contributions 3,4), lung (Contribution 5), liver (Contribution 6) and brain (Contributions 7–9). Finally, therapeutic approaches based on gut microbiota modulation are discussed by Contributions 10–14.

## 2. Development of Reliable Methods for Studying Human Microbiota

Contribution 1, by Plaza-Díaz and collaborators, investigated the optimal methods for studying the human microbiota, specifically focusing on fecal and breast samples from healthy women and breast cancer patients. The researchers compared the efficacy of three DNA isolation techniques (mechanical lysis, trypsin digestion, and saponin-based differential lysis) to minimize human DNA contamination, which often hinders microbial analysis. Additionally, they evaluated two sequencing approaches, *16S rRNA* gene sequencing and shotgun metagenomic sequencing, to identify discrepancies in the microbiome profiles obtained. The study concluded that for samples rich in microbial DNA, like feces, *16S rRNA* sequencing was most effective, while for tissues with complex microbial compositions, shotgun sequencing combined with the trypsin DNA extraction method offered the most accurate results.

## 3. Dynamics of Gut Microbiota Populations

Several reports have demonstrated that dominant gut microbial strains are unique to each individual and generally remain stable over time [4]. However, various conditions (e.g., treatment with antibiotics) may induce perturbations in these communities. After exposure to disruptive conditions, the dominant microbial strains usually recover, but the mechanisms involved in this process are not yet fully understood.

In Contribution 2, Koo & Morrow highlight that dominant fecal microbial strains tend to remain stable over time, despite antibiotic treatment. This study investigates the presence and dynamics of *Bacteroidales*-specific antimicrobial protein (BSAP) genes, specifically BSAP-2 (that lyses *Bacteroides uniformis* strains) and BSAP-3 (that lyses *Bacteroides vulgatus* strains), in human fecal microbial communities. This research utilized three publicly available longitudinal metagenomic datasets from healthy individuals and individuals treated with either a single antibiotic (cefprozil) or with multiple antibiotics (meropenem, gentamicin, and vancomycin). BLAST+ and Integrative Genomics Viewer tools were used for gene analysis. The findings showed that, following antibiotic treatment, the BSAP gene pattern from most individuals recovered to be the same as the pre-antibiotic strain. Although in a few individuals incomplete BSAP-3 genes were observed at early recovery times, they were replaced by *B. vulgatus* strains with complete BSAP-3 genes. These observations are consistent with the stability of *Bacteroides* strains over time and provide insights into the selection dynamics of dominant strains in the gastrointestinal tract following perturbation.

## 4. Skin Microbiota and Inflammatory Skin Diseases

The skin microbiota is gaining attention as a potential contributor to inflammatory skin diseases [5,6]. Indeed, skin and gut microbiota dysbiosis can influence skin inflammation through activation of immune pathways [7]. Two contributions focus on the relationship between skin inflammation and microbiota composition.

The preliminary study by Ferček et al. (Contribution 3) investigated the bacterial communities on the skin around the eyes (periocular region), comparing healthy individuals to those with inflammatory skin conditions like atopic dermatitis, seborrheic dermatitis, rosacea, and contact dermatitis. The researchers collected skin swabs to extract DNA, which was further analyzed to identify and quantify the different bacteria present, aiming to understand if specific microbial imbalances contribute to these diseases. Key findings included a significant difference in bacterial diversity between healthy and affected skin, with certain bacterial genera like *Rothia, Corynebacterium, Bartonella* and *Paracoccus* being more prevalent in patients with specific conditions, while others like *Anaerococcus*, *Bacteroides, Porphyromonas,* and *Enhydrobacter* were more abundant in healthy controls. The study suggests that changes in the periocular skin microbiota could play a role in the development of inflammatory skin diseases. The development of new diagnostic tools or targeted therapies that modify the microbiota to restore skin health or prevent disease progression may be assisted by an understanding of these microbial differences.

Contribution 4 (study by Chung et al.) investigated the frequency of dendritic epidermal T cells (DETCs)—a γδ T cell population residing in the skin—in mice under different housing conditions. The researchers observed that local skin inflammation significantly increased DETC numbers in Balb/c mice and that housing conditions impacted DETC populations. Exposure to bedding from a non-barrier environment led to higher DETC counts and distinct gut microbial compositions, notably an increase in *Bacillaceae* and a decrease in *Prevotellaceae*, which are known to influence T cell populations. These findings suggest a strong connection between the abundance of DETCs and the gut microbial composition, environmental stimuli, and the dynamics of skin immunity.

## 5. Gut–Lung Axis—Connection Between Gut Microbiota and Lung Diseases

Gut microbiota has been associated with the development of lung immunity [8,9]. Indeed, lungs and intestines share multiple epithelial structures, like goblet cells and IgA secretion. Perturbations of gut microbiota in early life lead to long-term deficient lung immunity [10], which demonstrates the importance of the gut–lung axis.

The comprehensive review by Alswat A.S. (Contribution 5) explores the profound connection between the gut microbiota and overall host health, with a particular focus on the gut–lung axis. This review highlights how the complex ecosystem of microorganisms in our gut plays a role in susceptibility to various respiratory conditions like asthma, cystic fibrosis, chronic obstructive pulmonary disease, and lung infections. It emphasizes that a balanced gut microbiota, a condition known as “eubiosis,” is vital for well-being, while an imbalance, or “dysbiosis,” can contribute to numerous diseases. Furthermore, the study addresses promising therapeutic approaches targeting the gut microbiota such as dietary interventions, the use of probiotics, prebiotics, symbiotics, and postbiotics, and fecal microbiota transplantation aimed at restoring this crucial microbial balance for improved respiratory health outcomes.

## 6. Liver Diseases and Bile Microbiota

Bile secretion, considered sterile in the past, has been shown to harbor a diverse microbial community, differing between healthy and pathological conditions of the biliary tract [11,12].

Lee et al. (Contribution 6) investigated the possible role of the bile microbiome and metabolites in cholangiocarcinoma (CCC) development, a type of bile duct cancer with high mortality. The authors collected bile samples from patients with and without CCC to analyze microbial composition and metabolic profiles, discovering that patients with CCC exhibited lower microbial diversity and a higher abundance of *Escherichia coli*. Notably, the study also identified significantly lower levels of isoleucine in the bile of CCC patients. Further in vitro experiments revealed that isoleucine suppressed CCC cell proliferation, suggesting its potential as a biomarker and therapeutic target for this challenging disease.

## 7. Neurological Disorders and the Gut–Brain Axis

The gut–brain axis represents a crucial bidirectional communication pathway between the central nervous system, the enteric nervous system, and the brain’s emotional and cognitive centers [13]. Three review reports (Contributions 7–9) indicate its pivotal role in numerous neurological diseases.

In Contribution 7, Nakhai and collaborators present an extensive review on the microbiota–gut–brain axis. The text systematically details how dysbiosis is implicated in conditions ranging from developmental disabilities, like Rett syndrome and autism spectrum disorder, to neurodegenerative diseases such as Alzheimer’s, Parkinson’s, and Huntington’s, as well as mental health conditions like depression and schizophrenia. It further investigates how therapeutics, diet, lifestyle factors, and early life experiences can, in turn, impact the interactions within the gut–brain axis, offering potential avenues for microbe-based interventions to improve patient outcomes.

Che Mohd Nassir et al. (Contribution 8) explored the importance of the gut–brain axis in general, with a particular focus on its role in the context of cerebral small vessel disease (CSVD). The authors describe potential links between gut microbiota and CSVD, highlighting how imbalances in gut bacteria contribute to neuroinflammation, vascular dysfunction, and impaired waste clearance in the brain. The paper also introduces the glymphatic system, the brain’s waste removal pathway, and the role of circulating cell-derived microparticles as key mediators in these complex interactions. Ultimately, the review suggests that modulating the gut microbiota through interventions like probiotics and diet could offer novel therapeutic avenues to improve brain health and mitigate the progression of CSVD.

Contribution 9 discusses the emerging role of the gut microbiota in mood disorders. This review article describes how dysbiosis contributes to conditions such as bipolar disorder and major depressive disorder by affecting neurotransmitters and inflammation. Finally, it reviews current and future therapeutic approaches including dietary interventions, prebiotics, psychobiotics (probiotics that confer mental health benefits), and fecal microbiota transplantation, highlighting the need for personalized medicine and further rigorous research to develop effective, targeted treatments for mood disorders.

## 8. Therapeutic Strategies Based on Microbiota Modulation

Administration of probiotics/postbiotics and dietary supplements was demonstrated to be beneficial for the management of gut and systemic cancers and inflammatory diseases [14,15]. Several contributions of this Special Issue addressed the therapeutic potential of different formulations.

Zhong et al. (Contribution 10) explored the use of postbiotics in colorectal cancer treatment. Postbiotics are substances produced by probiotic species and so postbiotic formulations do not contains living organisms [16]. The authors used a mouse model to investigate the inhibitory effects of administered *Weizmannia coagulans* MZY531 postbiotics on colorectal cancer growth. Evaluation of tumor growth, body weight, and various biochemical and cellular markers was performed. The results demonstrated that *W. coagulans* MZY531 postbiotics significantly inhibited tumor growth, reduced tumor size, and lowered serum levels of tumor markers. Multiple mechanisms of action against colorectal cancer were activated by the postbiotic treatment. The effects of postbiotics were often more pronounced than those observed with probiotics.

In Contribution 11, Ghannoum et al. demonstrated the ability of a probiotic–amylase blend to modulate gut microbiota, leading to a significant increase in beneficial *Saccharomyces cerevisiae* and a decrease in potential pathogens like *Bacillus thuringiensis* and *Macrococcus caseolyticus*. The authors hypothesized that these changes are possibly related to the improvement of clinical gastrointestinal symptoms observed in a previous study using the same cohort.

The study from Ko et al. (Contribution 12) aimed to investigate the effects of red beet powder and betanin pigment on the composition and metabolic activity of human gut microbiota using an in vitro gastrointestinal digestion and fecal fermentation model. Fecal samples from healthy subjects were used, which were classified into three enterotypes: *Phocaeicola*, *Prevotella*, and *Bifidobacterium*. The results suggest that beetroot powder and betaine pigment have enterotype-specific responses in the gut microbiota and SCFA production. S3-*Bifidobacterium* enterotype subjects showed the most significant decrease in alpha diversity (Chao index) and a different trend in other microbial changes as compared to the other enterotypes.

Treatment with probiotics may also be used to ameliorate liver function. Hizo & Rampelotto (Contribution 13) systematically reviewed the impact of the probiotic *Bifidobacterium* on various liver diseases and gut microbiota, including studies that used Next-Generation Sequencing (NGS) technologies for microbiota analysis. The core purpose was to evaluate *Bifidobacterium’s* potential as a safe and effective treatment by restoring intestinal microbiota balance, reducing inflammation, and improving clinical parameters in conditions like Non-Alcoholic Fatty Liver Disease, Alcoholic Liver Disease, and Cirrhosis. *Bifidobacterium* proved highly effective in restoring the balance of gut microbiota in humans and animals, leading to improved clinical and biochemical parameters in these diseases. A significant gap in the literature regarding *Bifidobacterium* efficacy in Non-Alcoholic Steatohepatitis and Hepatocellular Carcinoma was identified in this study, particularly concerning NGS-based research.

In Contribution 14, Mutlutürk and collaborators reported a clinical study exploring how a specially designed dietary intervention influenced the gut microbiota and various health markers in individuals with rheumatoid arthritis. The study found that adhering to this “Ideal Food Pyramid,” which differed from the standard Mediterranean diet by emphasizing specific vegetables, gluten-free grains, and supplements while avoiding salt and sugar, led to positive clinical benefits. These benefits included improvements in anthropometric measurements, disease activity, and certain biochemical parameters, alongside beneficial shifts in the gut microbiome composition by decreasing harmful bacteria like *Prevotella copri* and increasing beneficial ones such as *Faecalibacterium prausnitzii*. This suggests that targeted dietary approaches can serve as a valuable complementary strategy for RA management.

## 9. Conclusions

This Special Issue illustrates the important role of the human microbiota in maintaining health and influencing the development and outcome of numerous diseases, from localized conditions like inflammatory skin diseases to systemic conditions affecting the liver, brain, and overall gut function. The body of evidence presented here, largely enabled by recent sequencing technologies, offers deeper insights into the intricate mechanisms of host–microbe interactions.

While significant advances have been made in recent years, particularly in identifying microbial signatures and the efficacy of various interventions, the field still requires more robust and comprehensive research. Future studies must focus on larger cohorts, long-term longitudinal analyses and investigation of causal relationships and mechanisms of pathology, moving beyond simple correlations. The development of personalized microbiota-based interventions, tailored to an individual’s unique microbial profile, genetic trends and lifestyle, is a promising avenue for more effective therapies.

A deeper insight into the therapeutic potential of the human microbiota necessitates a multidisciplinary approach, integrating microbiology, nutrition, bioinformatics, and clinical science. This Special Issue demonstrates the dynamic and rapidly evolving nature of microbiome research, paving the way for revolutionary strategies in disease prevention, diagnosis, and treatment.

## Abbreviations

The following abbreviations are used in this manuscript:

BSAP	*Bacteroidales*-specific antimicrobial proteins
CCC	cholangiocarcinoma
CSVD	cerebral small vessel disease
DETC	dendritic epidermal T cells
NGS	Next-generation sequencing
